# Genome-wide analysis of SET-domain group histone methyltransferases in apple reveals their role in development and stress responses

**DOI:** 10.1186/s12864-021-07596-0

**Published:** 2021-04-19

**Authors:** Wenjie Li, Jinjiao Yan, Shicong Wang, Qianying Wang, Caixia Wang, Zhongxing Li, Dehui Zhang, Fengwang Ma, Qingmei Guan, Jidi Xu

**Affiliations:** 1 State Key Laboratory of Crop Stress Biology for Arid Areas/Shaanxi Key Laboratory of Apple, College of Horticulture, Northwest A&F University, Yangling, 712100 Shaanxi China; 2 College of Forestry, Northwest A&F University, Yangling, 712100 Shaanxi China

**Keywords:** *Malus×domestica*, Histone methylation, SET-domain group, Plant development, Stress response

## Abstract

**Background:**

Histone lysine methylation plays an important role in plant development and stress responses by activating or repressing gene expression. Histone lysine methylation is catalyzed by a class of SET-domain group proteins (SDGs). Although an increasing number of studies have shown that SDGs play important regulatory roles in development and stress responses, the functions of SDGs in apple remain unclear.

**Results:**

A total of 67 SDG members were identified in the *Malus×domestica* genome. Syntenic analysis revealed that most of the *MdSDG* duplicated gene pairs were associated with a recent genome-wide duplication event of the apple genome. These 67 MdSDG members were grouped into six classes based on sequence similarity and the findings of previous studies. The domain organization of each MdSDG class was characterized by specific patterns, which was consistent with the classification results. The tissue-specific expression patterns of *MdSDGs* among the 72 apple tissues in the different apple developmental stages were characterized to provide insight into their potential functions in development. The expression profiles of *MdSDGs* were also investigated in fruit development, the breaking of bud dormancy, and responses to abiotic and biotic stress; the results indicated that *MdSDGs* might play a regulatory role in development and stress responses. The subcellular localization and putative interaction network of MdSDG proteins were also analyzed.

**Conclusions:**

This work presents a fundamental comprehensive analysis of SDG histone methyltransferases in apple and provides a basis for future studies of MdSDGs involved in apple development and stress responses.

**Supplementary Information:**

The online version contains supplementary material available at 10.1186/s12864-021-07596-0.

## Background

Apple (*Malus×domestica*) is one of the most important fruit tree crops globally [1]. Histone lysine methylation plays an important role in plant development and stress responses by regulating gene expression and is catalyzed by a class of SET-domain group proteins (SDGs). Although an increasing number of studies have examined the genes controlling histone methylation, studies of the SDG histone methyltransferases involved in apple development and stress responses have been limited. Given that SDG proteins play important regulatory roles in plant development and stress responses, there is a need to characterize their roles in apple.

In eukaryotes, the nucleosome is composed of 147 base pairs of DNA wrapped around the histone octamer, which consists of two molecules each of the four types of histone proteins: H2A, H2B, H3, and H4 [2, 3]. The N-terminal region of the core histones (also called the “histone tail”) is covalently modified by various post-translational modifications, including acetylation, methylation, phosphorylation, ubiquitination, and sumoylation [4, 5]. These modifications can regulate gene expression by affecting chromatin structure and accessibility [6, 7].

Histone lysine methylation is one of the most well-studied histone modifications in plants and plays a fundamental regulatory role in plant growth and development, the reproductive process, and the response to environmental factors [7–10]. Histone H3 can be mono-, di-, or tri-methylated on K4, K9, K27, and K36 sites; H3K4 and H3K36 are transcription activation marks, whereas H3K27 and H3K9 are repression marks [8]. Histone lysine methylation is dynamically controlled by antagonistic histone methyltransferases (HMTs) and histone demethylases (HDMs), which are also called the “writers” and “erasers” of histone methylation [11–13]. The SET-domain group (SDG) protein family is the only known group of HMTs in plants.

SDG proteins were first discovered in *Drosophila melanogaster* and were named after the catalytic domain of Suppressor of variegation 3–9 (Suv), Enhancer of Zeste [E(z)], and Trithorax (Trx) [14]. Previous research has revealed that *Arabidopsis*, rice, and maize have 46, 34, and 31 SDGs, respectively, and they were further classified into seven groups [15]. Different SDG classes are responsible for catalyzing the methylation of different lysine sites. Generally, E(z), Suv, and ATXR5/6 proteins control H3K9 or H3K27 methylation leading to gene repression, whereas the Ash and Trx proteins catalyze H3K4 and H3K36 methylation resulting in gene activation [16].

In addition to several studies that have documented the critical role of *SDG* genes in plant developmental processes, histone methylation and *SDG* genes have also been shown to play a role in stress responses [6, 7, 17–20]. Previous studies have revealed that the histone methyltransferases MEA and SWN play an important role in *Arabidopsis* seed development, dormancy, and germination [21, 22]. Numerous studies have revealed that SDGs regulate the flowering process by modulating the histone methylation levels of flowering-regulatory genes. For example, ATX1 and ATX2 maintain the H3K4me3 deposition at the *FLC* locus to regulate flower time in *Arabidopsis* [23]. In addition, the repressive methylation mark H3K27me3, which is mediated by the CLF-containing PRC2 complex, mediates the repression of *FLC* and *FLC* relatives in the flowering process [23]. SDGs also play crucial roles in reproductive development. SDG4/ASHR3, a writer of H3K3me and H3K36me, regulates pollen tube growth and stamen development [24]. SDG8/ASHH2 is required for both anther and ovule development [25].

A genome-wide analysis of the H3K4me3 level associated with transcriptome data in rice under drought stress indicated that H3K4me3 deposition affects the expression levels of drought-responsive genes [26]. The enrichment of H3K4me3 and H3K9ac on the drought-inducible genes *RD29A* and *RD20* was observed under strong drought stress in *Arabidopsis* [27]. The Trx group protein ATX1, which catalyzes the H3K4me3 modification, can activate the ABA biosynthesis gene *NCED3* and enhance drought tolerance in *Arabidopsis* [28]. *Arabidopsis ATX4* and *ATX5* have been recently shown to play essential roles in ABA and dehydration responses [29]. Moreover, the histone methyltransferase SDG8 mediates epigenetic modifications on light, carbon, and brassinosteroid responsive genes and is involved in shoot branching and necrotrophic fungi defense [30–33]. These results indicate that SDGs modify histone methylation status on stress-responsive genes to regulate their expression in response to abiotic stresses.

In this study, a total of 67 SDG members were identified in the apple genome. Analyses of phylogenetic and syntenic relationships, domain organization, and gene structure were conducted. Moreover, the expression patterns of *MdSDGs* were investigated in the 72 dissected tissues collected during different apple growth and development stages. The expression profiles of *MdSDGs* were analyzed in different fruit development stages, at the breaking of bud dormancy, and under abiotic and biotic stress responses. Generally, this work presents a comprehensive analysis of SDG genes in apple and provides a basis for future work to explore the regulatory roles of *MdSDGs* in apple development and stress responses.

## Results

### Identification and syntenic analysis of SDG proteins in apple

**Fig. 1:**
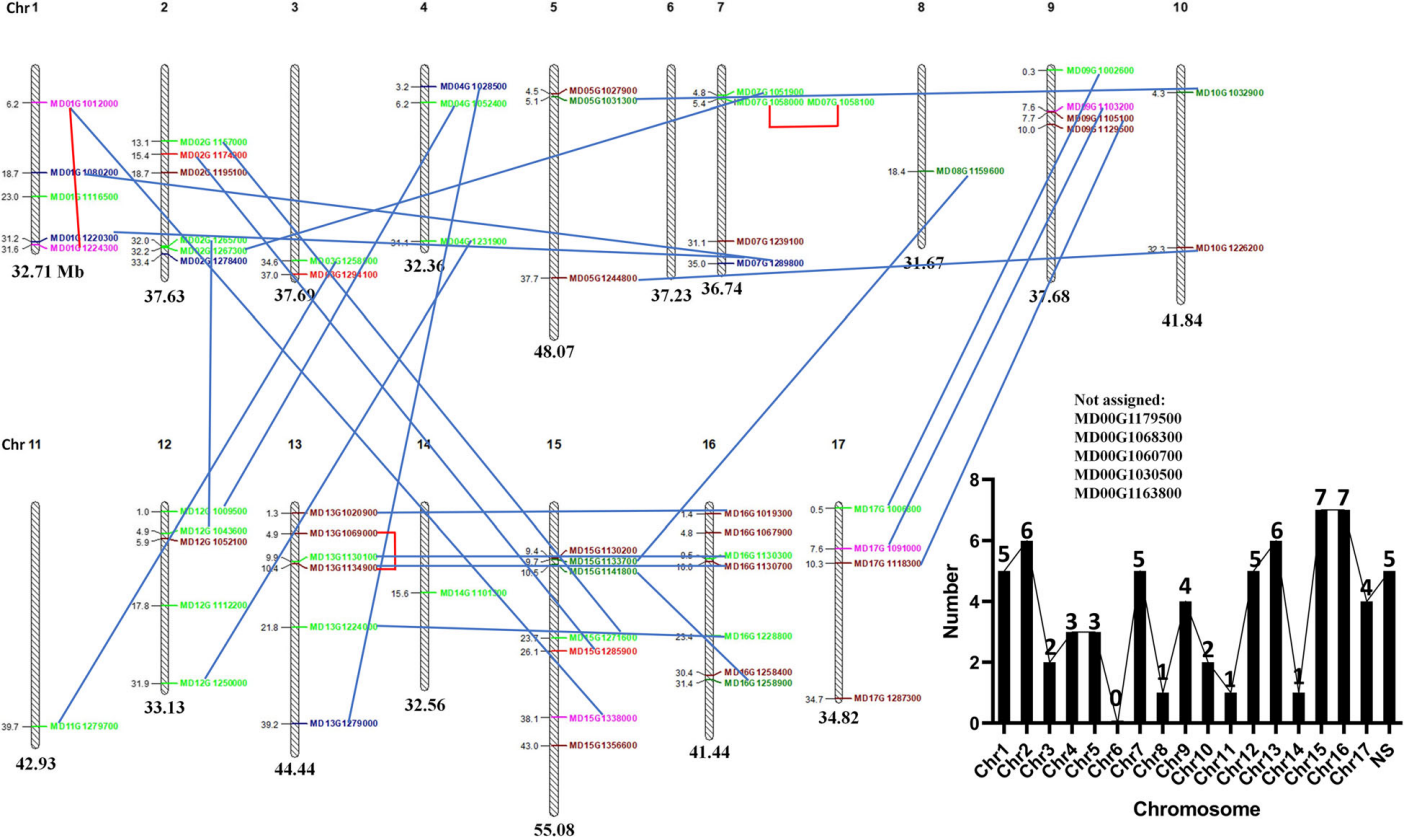
Chromosomal localization of *SDGs* in the apple genome. The *MdSDGs* found on duplicated chromosomal segments are connected by lines. The blue lines represent segmentally duplicated gene pairs, and red lines represent tandem duplicated gene pairs in the apple genome. The numbers of *MdSDGs* assigned to each chromosome are shown in the histogram

A total of 67 SDG members were identified in the apple genome, and they were further assigned to the 17 apple chromosomes; five of them could not be assigned (Fig. 1). The apple *SDGs* were distributed among the 17 chromosomes, except for chromosome 6. Chromosomes 15 and 16 contained the maximum number of 7 *MdSDG* members (Fig. 1). The duplication events among *MdSDG* members are connected by lines, and the tandem duplicated gene pairs are marked by red lines (Fig. 1). According to the previous study, the duplicated *MdSDGs* correspond to chromosome duplications, suggesting that the *MdSDG* duplications are accompanied by genome-wide duplication (GWD) events in the apple genome [34]. Moreover, the gene features of MdSDG members are displayed in Table S1.

**Fig. 2:**
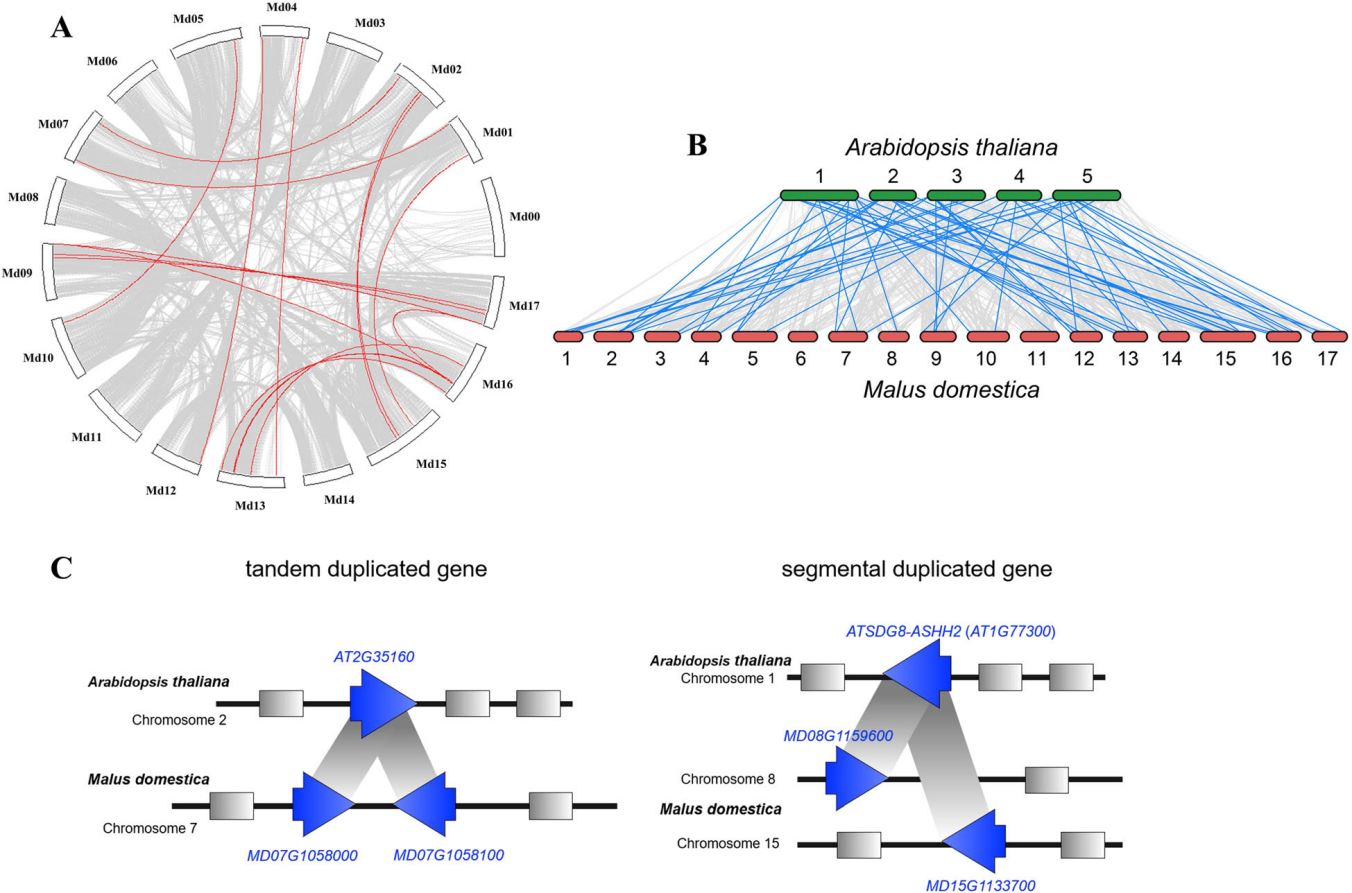
Syntenic analysis of *SDGs* in *Malus×domestica* and *Arabidopsis thaliana*. **a** *SDGs* in the same group are linked in red, and genes with no clear syntenic counterparts are linked by grey lines. **b** The syntenic analysis of *SDGs* between apple and *Arabidopsis*. The collinearities of *SDGs* are marked by blue lines. **c** The examples of tandem duplicated genes and segmentally duplicated genes among *Arabidopsis* and apple, respectively

To further explore the synteny and collinearity of *MdSDGs*, MCScanX (Multiple Collinearity Scan Toolkit) was used to perform the syntenic analysis among *MdSDG* members. The evolutionary analysis of *MdSDG* synteny and collinearity in the apple genome is shown in Fig. 2a. The 29 *MdSDG* gene pairs were identified as duplicated genes, and their divergence time was estimated (Table 1). Among them, three *MdSDG* gene pairs appear to represent a tandem duplication. The MdSDG gene duplication events correspond to a recent GWD event in *Malus*, which formed the apple 17-chromosome karyotype [34]. For example, one member of the duplicated gene pair is located in chromosome 5, the other is located in chromosome 10 (same as chromosome pairs 3–11, 9–17, 13–16) (Table 1). Most of the MdSDG duplicated gene pairs were derived from chromosome duplication events in the apple genome [34], suggesting that the MdSDG duplications were largely related to apple GWD events. Moreover, 29 duplicated gene pairs were used to calculate Ka (nonsynonymous substitutions per site), Ks (synonymous substitutions per site) [35], and the divergence time [36] (Table 1). All of the Ka/Ks values of paralogous MdSDGs gene pairs ranged from 0.07 to 0.94. As Ka/Ks < 1 generally indicates purifying or negative selection [37], suggesting that these gene pairs have undergone purifying or negative selection. Previous studies have revealed that values of Ks around 0.2 represent recently duplicated genes, and Ks around 1.6 represent paleo-duplication events [34]. In this study, the Ks of most of the MdSDG duplicated pairs ranged from 0.1–0.3, indicating that they are recently duplicated genes. However, the Ks of MD00G1060700/MD02G1265700 and MD01G1080200/MD07G1289800 duplicated gene pairs was 1.74 and 1.58, respectively, indicating that these two duplicated gene pairs were caused by paleo-duplication (Table 1) [34]. The syntenic relationships between the apple and *Arabidopsis* genomes are shown in Fig. 2b (SDGs are marked by blue lines) and reveal the expansion of the SDG family in apple (Fig. 2b). Examples of tandem duplicated genes and segmentally duplicated genes among *Arabidopsis* and apple are illustrated in Fig. 2c.

**Table 1:** The SDG duplicated gene pairs in apple

Syntenic gene pair		Ka	Ks	Ka/Ks	EffectiveLen	AverageS-sites	AverageN-sites	Divergence Time (MYA)
MD00G1060700	MD02G1265700	0.59	1.74*	0.34	2115	472.25	1642.75	58.15
MD00G1068300	MD12G1112200	0.02	0.12	0.19	2112	504.00	1608.00	4.16
MD00G1179500	MD03G1294100	0.04	0.18	0.22	1872	419.00	1453.00	5.98
MD01G1012000	MD15G1338000	0.03	0.17	0.19	1143	263.58	879.42	5.65
MD01G1080200	MD07G1289800	0.36	1.58*	0.23	3018	657.33	2360.67	52.62
MD01G1220300	MD07G1289800	0.04	0.17	0.22	3222	713.25	2508.75	5.51
MD02G1157000	MD15G1271600	0.04	0.15	0.28	4506	1002.00	3504.00	5.01
MD02G1174900	MD15G1285900	0.02	0.33	0.07	474	112.42	361.58	10.99
MD02G1267300	MD07G1051900	0.04	0.13	0.31	2061	476.42	1584.58	4.20
MD03G1258900	MD11G1279700	0.17	0.25	0.66	888	199.08	688.92	8.47
MD04G1028500	MD13G1279000	0.02	0.13	0.17	1557	365.50	1191.50	4.34
MD04G1231900	MD12G1250000	0.10	0.34	0.31	744	165.83	578.17	11.23
MD05G1031300	MD10G1032900	0.03	0.18	0.17	777	169.33	607.67	5.98
MD05G1244800	MD10G1226200	0.12	0.41	0.30	567	121.33	445.67	13.79
MD08G1159600	MD15G1133700	0.06	0.12	0.51	6240	1406.92	4833.08	4.07
MD09G1002600	MD17G1006800	0.02	0.14	0.17	2028	471.08	1556.92	4.74
MD09G1103200	MD17G1091000	0.03	0.14	0.26	7383	1669.42	5713.58	4.51
MD09G1129500	MD17G1118300	0.25	0.52	0.48	1212	288.67	923.33	17.35
MD12G1009500	MD04G1052400	0.79	NaN	NaN	1014	240.58	773.42	–
MD12G1043600	MD02G1265700	0.09	0.16	0.58	426	99.83	326.17	5.38
MD13G1020900	MD16G1019300	0.03	0.15	0.21	1431	338.25	1092.75	5.00
MD13G1130100	MD16G1130300	0.04	0.17	0.24	2010	462.42	1547.58	5.77
MD13G1134900	MD16G1130700	0.06	0.28	0.22	1194	278.08	915.92	9.42
MD13G1224000	MD16G1228800	0.02	0.22	0.09	1890	448.67	1441.33	7.43
MD14G1101300	MD00G1068300	0.01	0.01	0.94	1521	362.25	1158.75	0.28
MD15G1141800	MD16G1258900	0.50	NaN	NaN	819	177.83	641.17	–
**MD01G1224300**	**MD01G1012000**	0.31	3.21	0.10	1044	234.92	809.08	107.01
**MD07G1058000**	**MD07G1058100**	0.39	0.86	0.46	1443	324.58	1118.42	28.58
**MD13G1069000**	**MD13G1134900**	1.06	2.89	0.37	1164	269.75	894.25	96.21

The bold *SDG* gene pairs represented tandem duplicated genes and the others were segmentally duplicated genes. The divergence time of *SDG* gene pairs was calculated by T = Ks/2r (*r =* 1.5 × 10 ^− 8^) [36]

### Phylogenetic analysis of SDG proteins

**Fig. 3:**
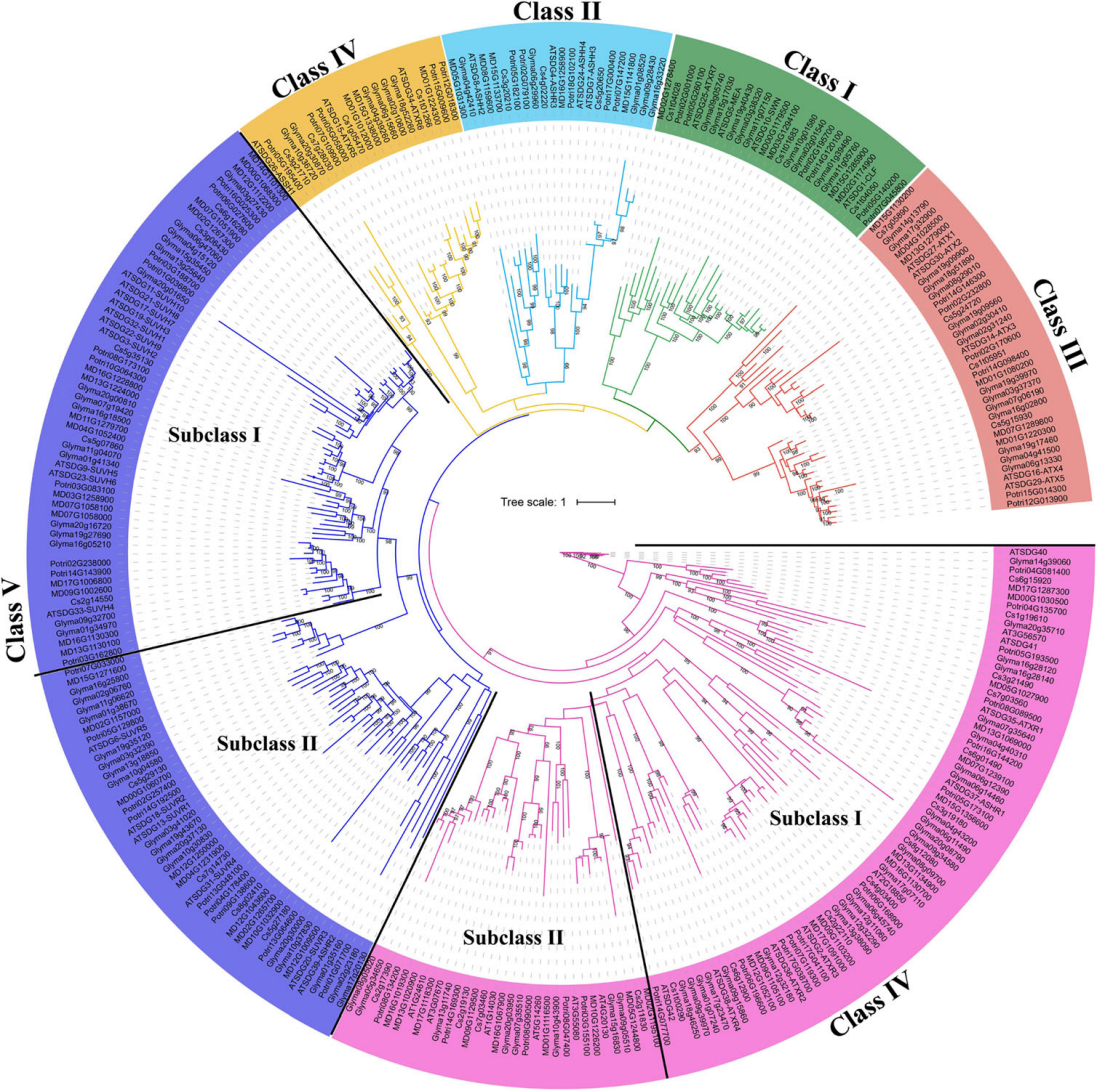
Phylogenetic analysis of SDGs. *Arabidopsis thaliana*, *Glycine max*, *Populus trichocarpa*, *Citrus sinensis*, and *Malus×domestica* SDGs were used for phylogenetic analysis; trees were constructed using the maximum likelihood method

SDG protein sequences from *Arabidopsis thaliana*, *Glycine max*, *Populus trichocarpa*, and *Citrus sinensis* were used to characterize the phylogenetic relationships of MdSDGs in apple (Fig. 3). The genomic IDs of SDG proteins are listed in Table S2. A total of 67 MdSDGs were clearly clustered into six groups: Class I, Class II, Class III, Class IV, Class V, and Class VI (Fig. 3). Class I contains 5 MdSDG members, and their counterparts in *Arabidopsis* are responsible for catalyzing H3K27 methylation [3]. Class II also has 5 MdSDGs, and Class II members are primarily responsible for H3K36 methylation [3]. A total of six MdSDG members belonging to Class III were assigned to the ATX family with H3K4 methyltransferase activity [38] (Fig. 3). Three MdSDGs of Class IV were homologous to ATXR5/6, which are known as H3K27 methyltransferases [39]. Class V contains 25 MdSDG members and is clustered into two subgroups including SUVH (subclass I) and SUVR (subclass II) proteins. Subclass I has 16 MdSUVH members, and Subclass II contains 9 MdSUVR members. SUVH and SUVR proteins are considered H3K9 methyltransferases according to previous studies [3, 40]. A total of 21 MdSDG members were assigned to Class VI and can be further clustered into two subgroups: subclass I contains SDGs with an interrupted SET domain and subclass II contains Rubis-subs-bind (RBS) proteins (Fig. 3). However, previous studies have reported that SDGs with an interrupted SET domain are likely involved in nonhistone protein methylation, and RBS proteins were identified as the subunit Rubisco methyltransferase [15, 41].

### Domain organization and gene structure of apple SDG genes

**Fig. 4:**
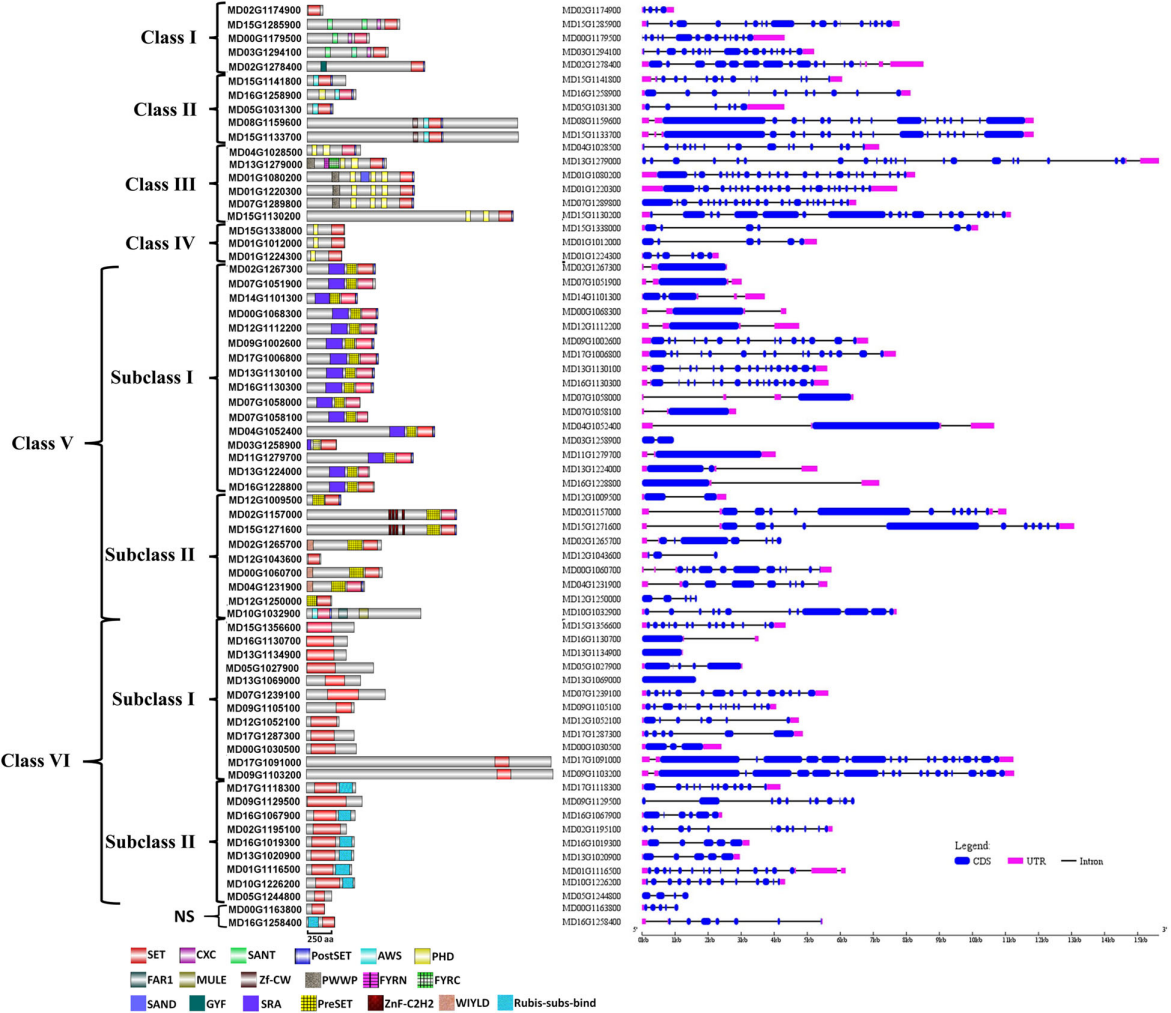
Domain organization (left) and gene structure (right) of MdSDG members. For domain architecture (left), the different color boxes represented different conserved domains. The conserved domains were as follows: SET (SM000317), CXC (SM001114), SANT (SM000717), PostSET (SM000508), AWS (SM000570), PHD (SM000249), FAR1 (PF03101), MULE (PF10551), Zf-CW (PF07496), PWWP (PF00855), FYRN (SM000541), FYRC (SM000542), SAND (SM000258), GYF (SM000444), SRA (SM000466), PreSET (SM000468), ZnF-C2H2 (SM000355), WIYLD (PF10440), and Rubis-subs-bind (PF09273). The gene structure (right) of *MdSDGs* includes CDS (blue box), intron (black line) and UTR (red box)

To explore the potential functions of MdSDGs, we further examined their domain architectures based on the SMART database. The domain organization of MdSDGs within the same class was relatively conserved (Fig. 4). The SET domain (SM000317) is generally located at the C-terminal of MdSDG proteins, except for Class VI members. Most Class I members are characterized by CXC (SM001114), SANT (SM000717), and SET domains. The SANT domain is the “SWI3, ADA2, N-CoR, TFIIIB” DNA-binding domain, which is present in many transcriptional regulatory factors and is essential for many chromatin-remodeling complexes [42]. This also suggests that Class I MdSDGs may play a role in chromatin regulation and the DNA-binding process. All Class II members are involved in AWS (SM000570), SET, and PostSET (SM000508) domain organization. Moreover, MD16G1258900 has an additional PHD (SM000249) at the N-terminal, and MD08G1159600/MD15G1133700 contains an additional Zf-CW (PF07496) domain. The AWS domain is associated with the SET domain and is involved in the methylation of histones and other proteins [43]. PostSET runs from the C-terminal to the SET domain and participates in S-adenosylmethine-binding and histone tail interactions [44]. However, Class III members are characterized by PWWP (PF00855), 2 or 3 copies of PHD (SM000249), SET, and PostSET domains, except that MD04G1028500 lacks the PWWP domain (Fig. 4). PWWP is named after a conserved Pro-Trp-Trp-Pro motif, which is involved in DNA binding and protein interaction [45]. PHD (plant homeodomain) plays an important role in epigenetic and chromatin-mediated transcriptional regulation [46]. Three Class IV members contain PHD and SET domains and are similar in their domain organization and protein lengths. Class V features two subgroups that are distinguished in their domain compositions, which coincides with the results of the phylogenetic analysis. Subclass I consists of SUVH proteins including SRA (SM000466), PreSET (SM000468), SET, and PostSET conserved domains. SRA is also termed SRA-YDG because the conserved YDG motif consists of the SET and Ring finger-associated domain [47]. PreSET is involved in maintaining the structural stability of SDGs [44]. All of the subclass II members lack the SRA domain, and three of them (MD02G1265700, MD00G1060700 and MD04G1231900) contain an additional WIYLD (PF10440) domain (Fig. 4). Class VI members were also clustered into two subgroups based on their domain compositions. All of the Subclass I members are interrupted SET domain proteins, and their functions are largely unknown in plants [48]. However, most Subclass II members have an additional Rubis-subs-bind (PF09273) at the C-terminal of the SET domain, except MD09G1129500 and MD05G1244800 (Fig. 4).

Furthermore, the conserved amino acids in the SET domain among the 67 MdSDGs were analyzed by WebLogo. Four conserved motifs were identified and contained conserved amino acid sites in Fig. S1. Specifically, glycine (G) at positions 254, 256, and 269 were highly conserved in motif 1 (243–270 aa). Motif 2 (290–315 aa) shows highly conserved glutamic acid (E) and glycine (G) at positions 303 and 306, respectively. Motif 3 (449–486 aa) displays conserved 460 asparagine (N), 461 Histidine (H), and 478 asparagine (N). In motif 4 (528–565 aa), the 559 tyrosine (Y) is highly conserved (Fig. S1). To explore the putative functional diversification of *MdSDGs*, gene structures with exon/intron constitutions are shown in Fig. 4. All of the *MdSDG* genes assigned from Class I to Class V contain intron/exon structures, and the numbers of exons vary greatly among *MdSDG* members. However, Class VI members *MD13G1134900* and *MD13G1069000* have only one exon and no introns. Most Class III members show complex exon/intron structures and contain the largest numbers of exons (Fig. 4).

### Expression analysis of MdSDGs in different tissues

To explore the potential functions of *MdSDGs* in different tissues, the tissue-specific expression patterns of *MdSDGs* in 72 dissected apple tissues were investigated (Fig. S2). The expression levels of *MdSDGs* in different tissues were extracted from the released transcriptome data (http://bar.utoronto.ca/efp_apple/cgi-bin/efpWeb.cgi). *MD00G1179500* and *MD03G1294100* of Class I showed high expression levels in fruit tissues during developmental stages, especially in the embryo tissues dissected from the fruits at 42 and 63 days after flowering (R48 and R53 stages in Fig. S2). *MD05G1031300* shows a higher expression level compared with other Class II members and is predominately expressed in embryo tissue, especially in the mature fruit stage (R62 and R68). However, *MD15G1133700* is preferentially expressed in anther (R23) and pollen (R28), and three members of Class III (*MD04G1028500*, *MD13G1279000*, and *MD01G1080200*) are also highly expressed in anther and pollen. *MD15G1338000*, *MD01G1012000*, and *MD01G1224300* of Class IV, as well as *MD09G1002600* and *MD17G1006800* of Class V, are weakly expressed in various tissues but are highly expressed in anther and pollen (Fig. S2). In Class VI, *MD16G1067900* is highly expressed in open-pollinated seedling tissues, particularly in the cotyledon, leaf, and leaf blade. Moreover, *MD13G1020900* of Class VI is highly expressed in various tissues, especially in the vegetative tissues in early stages and in seedling tissues (Fig. S2). These results provide new insight into the functional roles of *MdSDGs* in apple growth and development processes.

### Expression profiles of MdSDGs involved in apple development

**Fig. 5:**
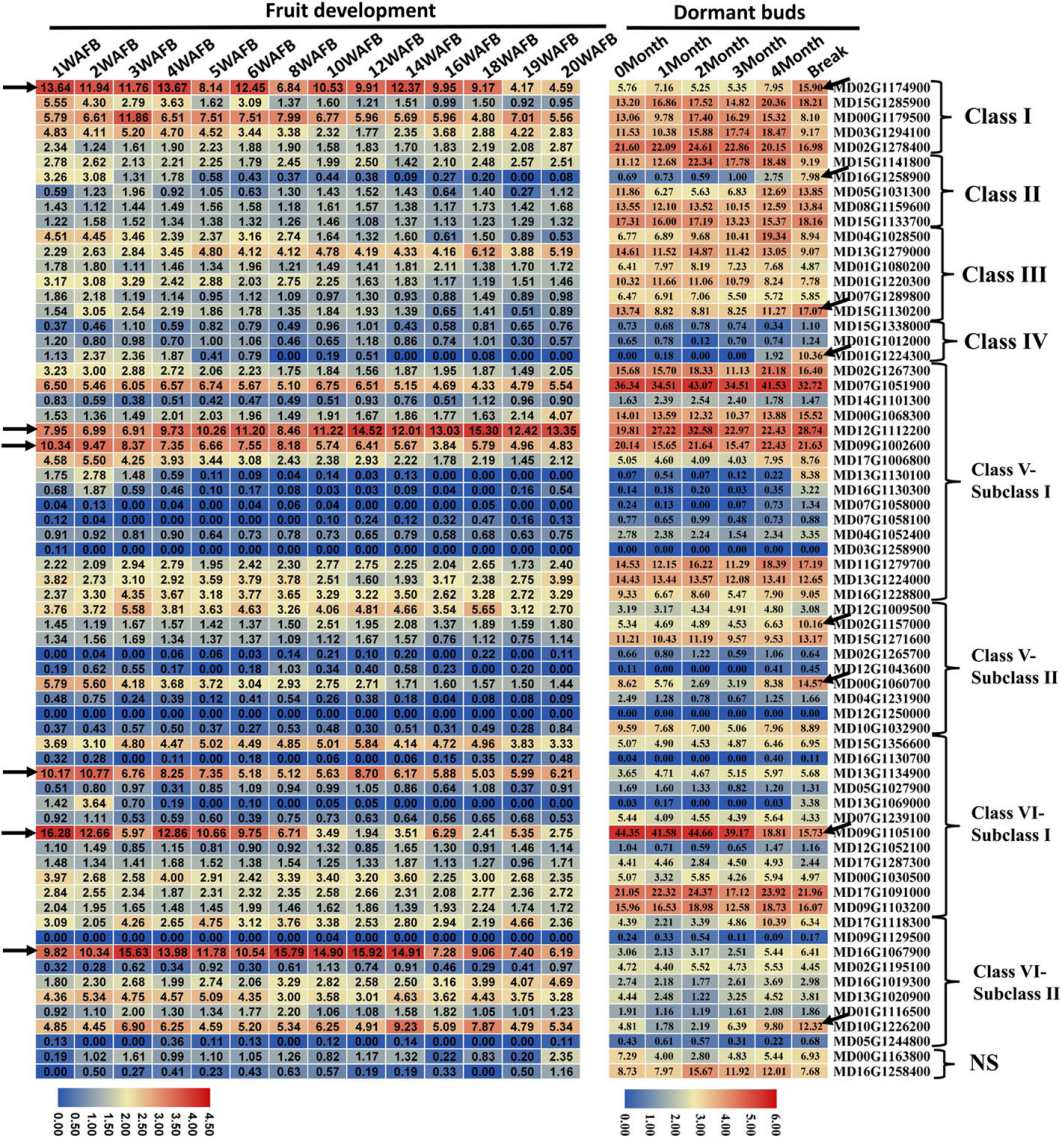
The expression profiles of *MdSDGs* in apple fruit development and the breaking of bud dormancy. The expression data were downloaded from the AppleMDO database (http://bioinformatics.cau.edu.cn/AppleMDO/index.php) based on previous studies and were illustrated with heat maps. The samples of these data were the fruit development stages with 1–20 weeks after full blooming (WAFB) (SRR3384922). The dormant buds were collected at 0, 1, 2, 3, and 4 months and the bud break stage (SRP099578). The log_2_ values of *MdSDGs* FPKM (Fragments Per Kilobase per Million) levels among different samples are used to reflect the gene expression levels and are displayed with numbers in each square

To gain insights into the biological role of *MdSDGs* in developmental processes, we analyzed their expression profiles during fruit development and dormant bud growth based on the AppleMDO database [49]. The expression levels of *MD02G11749000*, *MD15G1285900*, *MD09G1002600*, *MD13G1134900*, and *MD09G1105100* decreased during fruit development (Fig. 5), suggesting that these genes play regulatory roles in fruit development and ripening. In contrast, the expression of *MD12G1112200* increased with apple fruit development. *MD16G1067900* had the highest expression level in the middle of fruit development (Fig. 5). During dormant bud development, the mRNAs of *MD02G1174900*, *MD16G1258900*, *MD01G1224300*, *MD02G1157000*, *MD00G1060700*, *MD10G1226200*, and *MD15G1130200* are highly abundant during the stage in which dormancy is broken, suggesting that they play a role in regulating the breaking of dormancy (arrows in Fig. 5). The expression level of *MD09G1105100* decreased during the breaking of bud dormancy. These results provide insight into the potential functions of *MdSDGs* in fruit development and the breaking of dormancy.

### Expression patterns of MDSDGs in response to biotic and abiotic stresses

**Fig. 6:**
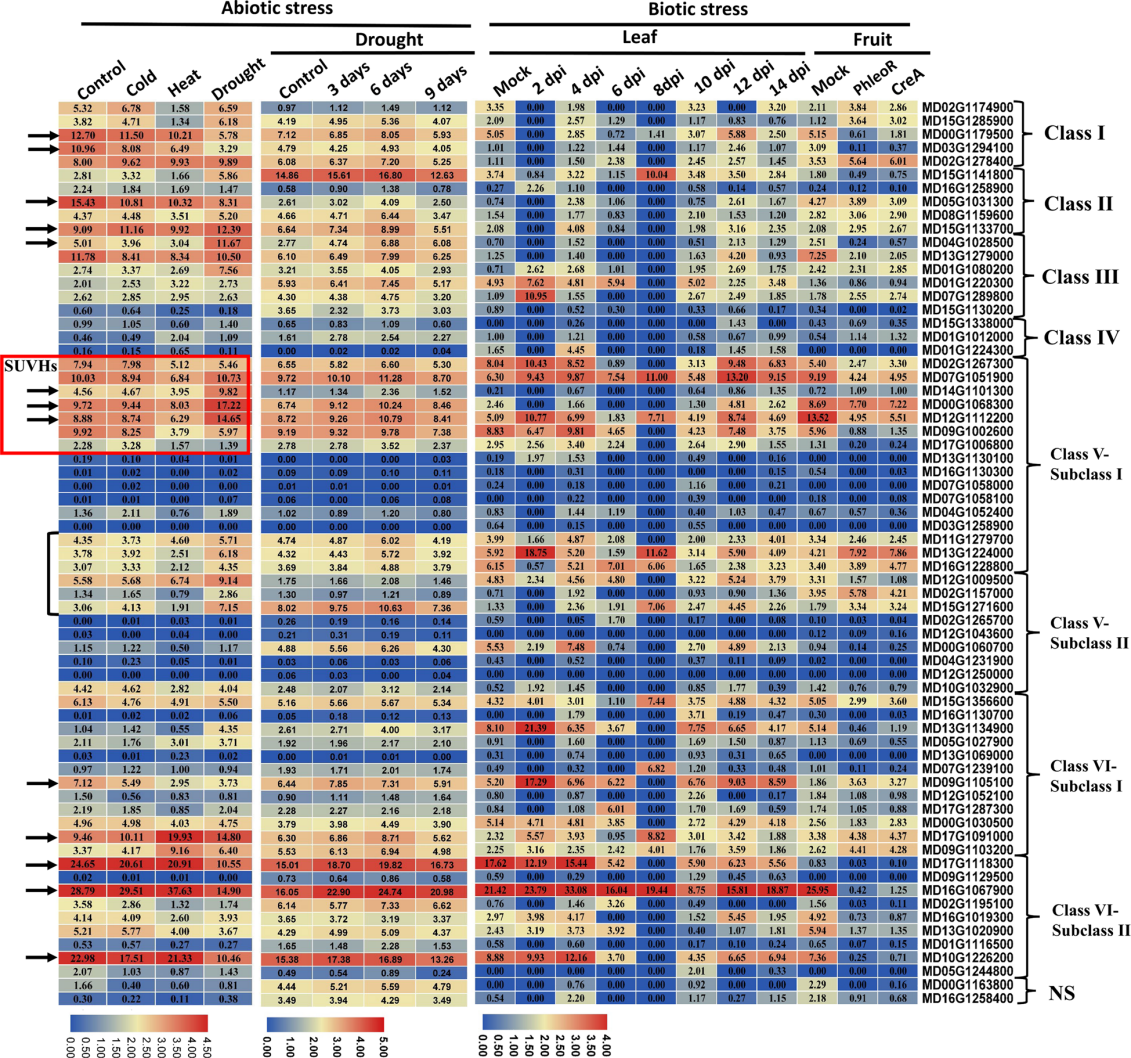
The expression profiles of *MdSDGs* in response to abiotic and biotic stresses. For the abiotic stresses, ‘Golden Delicious’ (*Malus×domestica*) seedlings were subjected to cold (3 h), heat (0.5 h), and drought (6 d) treatments, and the leaf samples were collected for RNA-seq analysis. Each abiotic treatment was performed with three biological replicates. For *Malus hupehensis* seedings under drought, samples used for transcriptome analysis were collected at 0, 3, 6, and 9 days. The biotic analysis data were downloaded from the AppleMDO database (http://bioinformatics.cau.edu.cn/AppleMDO/index.php) based on the previous studies and illustrated with heat maps. The samples of these data were leaves infected with *V. inaequalis* at 0–14 dpi (SRP018878) and mature fruit infected by *P. expansum* (SRP150975). The log_2_ values of *MdSDG* FPKM (Fragments Per Kilobase per Million) levels among different samples are used to reflect the gene expression levels and are displayed with numbers in each square

To explore the regulatory role of *MdSDGs* in response to biotic and abiotic stresses, we assessed the expression profiles of *MdSDG*s in response to various biotic and abiotic stresses (Fig. 6). The expression levels of *MdSDGs* were generally down-regulated in the early stages of *V. inaequalis* infection in leaves, especially at 2 and 8 dpi. During the following stages, the mRNA abundances of *MdSDGs* were partly recovered to the levels of the control stage (Fig. 6). Notably, several *MdSDGs* were dramatically up-regulated by *V. inaequalis* infection. For example, four genes including *MD07G1289800*, *MD13G1224000*, *MD13G1134900*, and *MD09G1105100* were markedly up-regulated by *V. inaequalis* infection at 2 dpi, and *MD15G1141800*, *MD15G1271600*, *MD07G1239100* and *MD17G1091000* were distinctly induced at 8 dpi (Fig. 6) [49]. Moreover, the expression levels of most *MdSDGs* were repressed in fruits infected by *P. expansum* (Fig. 6) [49]. However, small amounts of *MdSDGs* were up-regulated by the infection, such as *MD02G1278400*, *MD13G1224000*, *MD17G1091000*, and *MD09G1103200* (Fig. 6).

We also investigated the expression profiles of *MdSDGs* under cold, heat, and drought stresses. The expression data were obtained from the transcriptome data of ‘Golden Delicious’ seedlings under different abiotic stresses (SRS1872560). The mRNA abundances of *MD00G1179500* and *MD03G1294100* of Class I and *MD15G1133700* of Class II were distinctly suppressed under cold, heat, and drought treatments, especially drought stress (Fig. 6). However, *MD15G1133700* and *MD04G1028500* were up-regulated by drought. Similarly, the expression level of *MD17G1091000* was significantly induced by heat and drought stresses, especially heat stress. The expression levels of three genes, *MD14G1101300*, *MD00G1068300*, and *MD12G1112200* of Class V, were markedly induced under drought stress, suggesting that they played regulatory roles in the drought stress response. The expression of genes from *MD11G1279700* to *MD15G1271600* of Class V was highly induced in response to drought (Fig. 6). However, nearly half of the Class V members showed extremely low mRNA abundances and were thus considered non-expression genes (Fig. 6). Numerous Class VI genes were down-regulated under various abiotic stresses. For example, the expression levels of *MD09G1105100*, *MD17G1118300*, and *MD10G1226200* were inhibited by cold, heat, and drought stresses. However, *MD16G1067900* was up-regulated under heat stress and sharply down-regulated under drought stress (Fig. 6). To further characterize the expression changes of *MdSDGs* under different stages of drought treatment, we analyzed the expression patterns of *MdSDGs* at four stages of drought treatment. The results showed that the expression levels of most *MdSDGs* were up-regulated at 3 and 6 days of drought treatment and recovered at 9 days of drought treatment (Fig. 6).

**Fig. 7:**
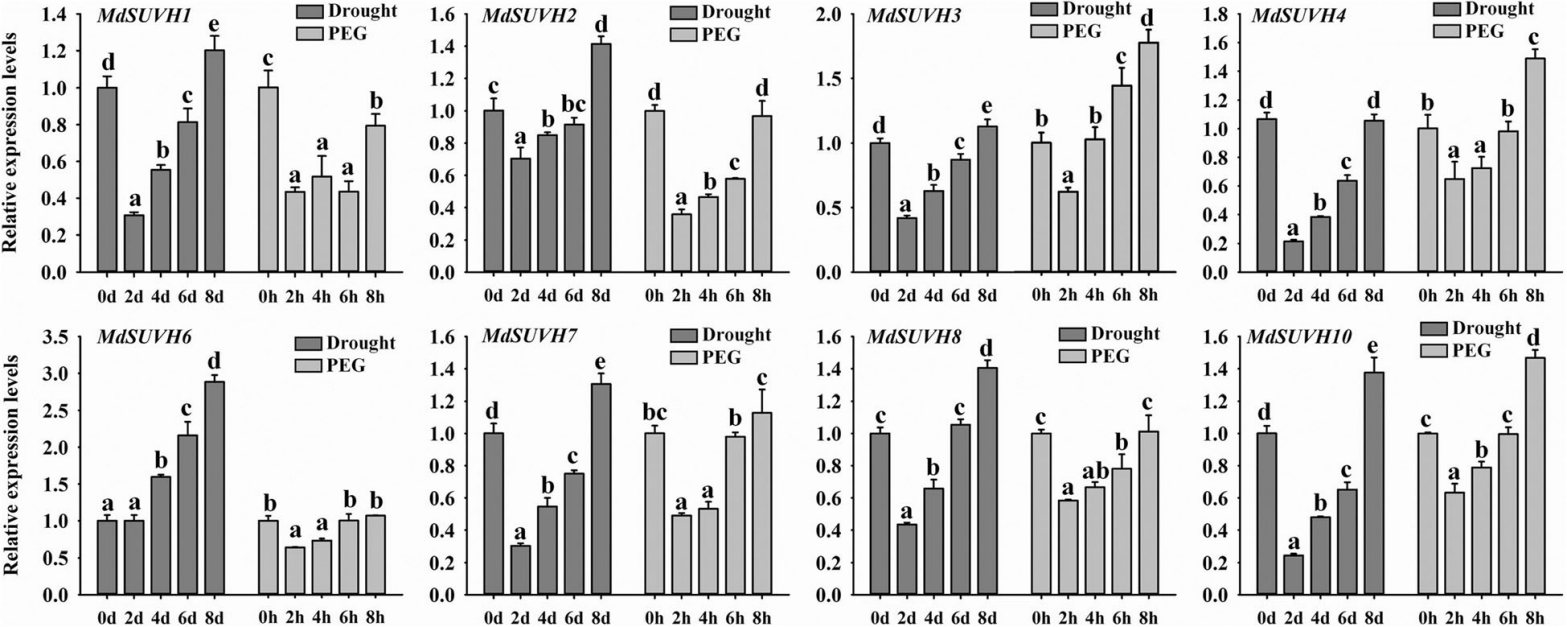
The relative expression levels of *MdSUVHs* in response to drought and PEG treatment. The grafted ‘Golden delicious’ plotted plants were under drought treatment for 0, 2, 4, 6, and 8 days. For PEG treatment, ‘Golden delicious’ seedlings were under 20% PEG for 0, 2, 4, 6, and 8 h. Three biological replicates were performed for each treatment in the qRT-PCR analysis. The apple malate dehydrogenase gene (*MdMDH*) was used as the reference gene. All qRT-PCR data were calculated as relative units after normalization to the reference gene, which was the internal control. The expression levels of the non-PEG treatment were used to calibrate the relative expression levels. Statistically significant differences were assessed using SPSS software based on Tukey’s test at *P* < 0.05. Different letters among samples indicate significant differences in expression

Based on the above results, we found that the *SUVH* histone methyltransferase genes responded to drought stress (the red box in Fig. 6). To investigate the potential functions of *MdSUVHs* in response to drought, we assessed the expression patterns of *MdSUVHs* in response to drought and PEG (polyethylene glycol) treatments using qRT-PCR. The changes in the expression of *MdSUVHs* under drought treatment were consistent with those observed under PEG treatment (Fig. 7). The expression patterns of *MdSUVH* members were similar under drought and PEG treatments, suggesting that they are functionally redundant in the response to drought stress. The expression levels of most *MdSUVHs* decreased rapidly at 2 days/hours, gradually recovered to the control level at 4 and 6 days/hours, and then became slightly higher than the control at 8 days/hours (Fig. 7). These results indicate that these *MdSUVH* members rapidly respond to water deficits in the early stages and possibly play a role in the drought response.

### Subcellular localizations and interaction networks of MdSDG proteins in apple

**Fig. 8:**
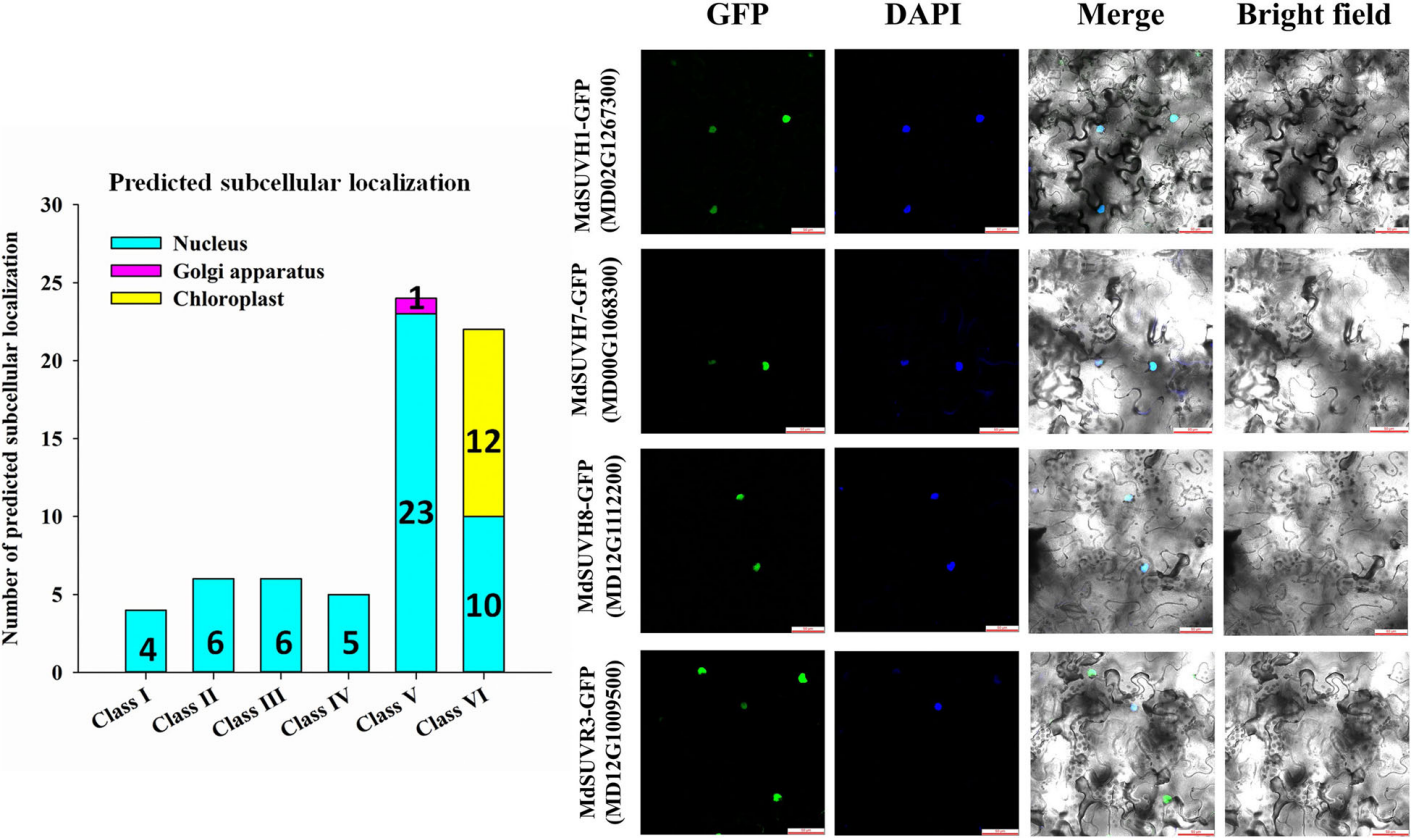
Subcellular localization analysis of MdSDGs. The predicted subcellular localizations of MdSDG proteins are listed in Table S1, and the numbers of each localization type are shown (left). The subcellular localizations of MdSUVH1, MdSUVH7, MdSUVH8, and MdSUVR3 were fused with GFP and analyzed in tobacco leaves. Nucleus were discerned using DAPI staining and false-colored in blue. Bar = 50 μm (red line in figure). The subcellular localization experiment was repeated three times, and at least three tobacco plants were infiltrated each time for each gene

We used Plant-mPLoc to predict the subcellular localization of MdSDGs. As expected, all Class I, Class II, Class III, Class IV, and Class V (except MD12G1250000) members were localized in the nucleus. Nearly half of the Class VI members were localized in the chloroplast, most of which were RBS proteins (Fig. 8). To further verify the subcellular localization results, we performed the subcellular localization analysis of MdSUVH1 (MD02G1267300), MdSUVH7 (MD00G1068300), MdSUVH8 (MD12G1112200), and MdSUVR3 (MD12G1009500) in tobacco. All of these proteins were localized in the nucleus, which is consistent with the predicted results (Fig. 8). The localization of MdSDGs in the nucleus was also consistent with their known functions in catalyzing the histone methylation process in the nucleus. Moreover, to explore the potential protein interactions among MdSDGs, we used STRING software to construct the interaction network among MdSDG proteins, and 40 MdSDGs were present in the interaction network (Fig. S3).

## Discussion

### The duplications of MdSDG gene pairs are related to apple GWD events

As the writers of histone lysine methylation, SDGs are responsible for adding methyl groups on histone lysine residues and are involved in many biological processes [3, 11]. As few studies have examined SDGs in apple, this study provides new insight into apple SDG members and explores their potential functions in plant development and stress responses. A total of 67 MdSDG members were identified from the apple genome in this study (Fig. 1). A total of 32 and 46 SDG proteins have been identified in *Arabidopsis thaliana* [15, 48]. A total of 31, 34, 51, 43, 33, and 40 SDG members have been identified in *Zea mays*, *Oryza sativa* [15], *Gossypium raimondii* [41], *Solanum lycopersicum* [50], *Vitis vinifera* [51], and *Citrus sinensis* [13], respectively. We found that the number of apple SDGs was greater than that in other plant species and nearly two-fold greater compared with grape and rice. According to the phylogenetic results (Fig. 3), one *Arabidopsis* SDG member generally corresponded to two apple homologs. All of these findings might relate to the GWD events in the apple genome. A previous study had demonstrated that the apple 17-chromosome karyotype of apple was derived from a 9-chromosome ancestor, and most MdSDG duplicated gene pairs corresponding to the chromosome duplication events in the apple genome, such as chromosome pairs 3–11 (they derived from one chromosome ancestor), 9–17, 13–16, and 4–12 [34]. Additionally, the values of Ks for the MdSDG duplicated pairs were around 0.2 (Table 1), suggesting that the duplicated genes were caused by recent GWD events [34]. In conclusion, the expansion of SDGs in apple might be largely related to the recent GWD events, along with the gene duplications.

### The specific domain composition contributes the multiple roles of SDGs

Epigenetic regulation plays a fundamental role in modulating gene expression, and how epigenetic factors recognize their target loci is an interesting topic and remains unclear. However, a growing body of recent work has uncovered the targeting mechanisms of these epigenetic factors [52]. For example, *Arabidopsis* SUVR5 contains C_2_H_2_ ZnF domains that cause SUVR5 to directly bind to target DNA and establish the heterochromatic state by H3K9me2 deposition in the target DNA [53]. The SUVR5 zinc finger domain acts like the “transcriptional factor” to bind specific DNA sequences that map to the promoters of target genes [53]. Similarly, *Arabidopsis* histone demethylase JMJ12 also possesses C_2_H_2_ ZnF domains, which can directly recognize a specific DNA motif (CTCTGYTY) and target specific genomic regions for gene activation [54]. These results indicate that the C_2_H_2_ ZnF domains contribute to the DNA binding ability of histone modifiers.

In this study, we characterized two C_2_H_2_ ZnF containing MdSDGs, MD02G1157000 and MD15G1271600 (Fig. 4), which are homologous to *Arabidopsis* SUVR5 (Fig. 3). Based on previous studies, these two SDGs may target specific genomic regions processing histone methylation by their DNA binding ability and modulate gene expression. Interestingly, their expression levels were induced by drought treatment (Fig. 6), suggesting that they play regulatory roles in the drought response [55]. Another interesting domain is the SRA domain in SUVH proteins (Fig. 4). Previous studies have shown that SRA domains are methyl-cytosine binding domains that vary in their sequence specificity [56]. For example, *Arabidopsis* SUVH2 SRA showed strong binding to methylated CG, but SUVH9 SRA showed a strong affinity for methylated CHH over CHG or CG sites, indicating the different roles of SUVH members [40, 56]. However, all of the subclass I MdSDGs of Class V contain the N-terminal SRA domain, indicating their methylated DNA binding specificity (Fig. 4).

### Characterizing the regulatory roles of histone modifiers aids the use of epigenome engineering for crop improvement

Currently, genomic selection and other molecular marker-based selection approaches are used for plant breeding, which rely on genetic variation. Although there is growing evidence that epigenetic regulation can also potentially contribute to crop improvement, the potential to apply epigenetics to crop improvement has received less attention [57, 58]. With the development of genome editing technology, new methodologies for engineering epigenetic modifications in a site-specific manner in plant genomes use genome-editing enzymes [58, 59]. The first study of site-specific epigenome engineering in plants was accomplished using a zinc finger nuclease (ZFN) fused to SUVH9 protein in *Arabidopsis* [47]. The SUVH9 protein is involved in the RNA-directed DNA methylation (RdDM) pathway and the ZFN-SUVH9 fusion can direct DNA methylation to target genomic loci and cause phenotypic changes [47]. With the rapid development of CRISPR-Cas9 systems for editing genome sequences, a valuable tool for the site-specific manipulation of plant epigenomes was made by fusing a nuclease-dead form of Cas9 (dCas9) and epigenetic regulators [60]. Specifically, they utilized a SunTag system by fusing dCas9 and the catalytic domain of DRM methyltransferase to target DNA methylation on the *FWA* promoter and trigger a stable developmental phenotype [59]. The success of site-specific DNA methylation editing indicates that we could fuse dCas9 and histone modifiers to specifically modulate histone modification levels of target genes for crop improvement. Therefore, uncovering the functions and regulatory mechanisms of histone modifiers in biological processes is fundamentally important for using epigenome engineering to promote crop improvement.

## Conclusions

In conclusion, this study provides new insight into SDG histone methyltransferases in apple. A total of 67 SDG members were identified in the apple genome, and they were distributed among the 17 chromosomes. Syntenic analysis indicates that most of the MdSDG duplicated pairs are associated with a recent GWD event in the apple genome. Phylogenetic analysis classified MdSDG proteins into six groups, and analyses of domain organization and gene structure were conducted. The tissue-specific expression patterns of *MdSDGs* among 72 apple tissues were characterized to explore the potential functions of these genes in different organs. The expression profiles of *MdSDGs* were investigated in fruit development, the breaking of bud dormancy, as well as the abiotic and biotic stress responses. Overall, this work presents a comprehensive analysis of SDG histone methyltransferases in apple, and the results will aid future efforts to use epigenome engineering for crop improvement.

## Methods

### Identification of MdSDGs from the sequenced apple genome

The sequences of MdSDGs were identified from the sequenced apple genome [61] by using HMMER v3.0 software (http://hmmer.org/). The HMM file of SDG conserved domain (PF00856), which was downloaded from Pfam database (http://pfam.xfam.org/), was used as a query to search apple genome (https://iris.angers.inra.fr/gddh13/, version 1.1). The identified MdSDGs were listed in Table S1 in detail, including genomic ID, chromosome position, protein length, and subcellular localization prediction. Then, all MdSDG protein sequences were submitted to SMART database (http://smart.embl-heidelberg.de/) to identify the conserved domains of MdSDGs. The default parameters were used to identify conserved domains with E < 0.01.

### Chromosome location, phylogenetic tree, domain organization, and gene structure analyses of MdSDG members

MapChart software [62] was applied to construct the chromosome location maps of MdSDGs according to the positions provided by genome database. MCScanX (Multiple Collinearity Scan Toolkit) software [63] was applied to analyze the syntenic relationships among SDG members in apple and *Arabidopsis*. The Ka and Ks values among MdSDG proteins were calculated by TBtools [35]. The divergence time (T) of syntenic gene pairs was evaluated by T = Ks/2r, *r* = 1.5 × 10^− 8^ [36]. The *Arabidopsis thaliana*, *Glycine max*, *Populus trichocarpa*, *Citrus sinensis* and *Malus×domestica* SDG proteins were used for phylogenetic analysis. The multiple alignment of above SDGs in five species was performed by MUSCLE software. Then, the ModelFinder was used to evaluate the best model (PMB + F + R7) for phylogenetic analysis [64] and the phylogenetic tree was constructed by IQ-TREE by using Maximum-likelihood method with 1000 ultrafast bootstrap replicates [65].

To investigate the domain composition of MdSDGs, the complete amino acid sequences of MdSDGs were subjected to SMART database, including outlier homologs and PFAM domains. The default parameter of SMATR database was applied to identify conserved domains. The output results were illustrated by the IBS 1.0.2 software (Illustrator for Biological Sequences) [66]. The gene structures of *MdSDGs* were displayed by Gene Structure Display Server (GSDS: http://gsds.cbi.pku.edu.cn/). To further explore the subcellular localization of MdSDG proteins, Plant-mPLoc (http://www.csbio.sjtu.edu.cn/bioinf/plant-multi/) was applied to predict the subcellular localization of MdSDGs. To investigate the conserved amino acids of SET domain, we aligned the SET domain amino acid sequences by Clustal Omega (https://www.ebi.ac.uk/Tools/msa/clustalo/) and then submitted to WebLogo (http://weblogo.berkeley.edu) to illustrate conserved amino acid sites (Fig. S1).

### Expression profiles of MdSDGs involving in development and stress responses

The expression levels of *MdSDGs* among different tissues were obtained from Apple eFP Browser database (http://bar.utoronto.ca/efp_apple/cgi-bin/efpWeb.cgi) and illustrated with heat maps (Fig. S2). Gene expression levels in Apple eFP Browser database were generated by RNA-seq data of 72 tissues and organs in different apple developmental stages which were performed by Prof. Steve van Nocker (Michigan State University). The details of sample collection and RNA-sequencing process were displayed in the website.

The expression patterns of *MdSDGs* involving in apple fruit and dormant bud development (Fig. 5), as well as apple in response to biotic stresses (Fig. 6) were extracted from AppleMDO database (http://bioinformatics.cau.edu.cn/AppleMDO/index.php) [49]. The original RNA-seq data of fruit development, dormant buds and biotic stress responses were collected in SRA database with SRR3384922 [67], SRP099578 (Foundation Edmund Mach) and (SRP150975) [68]. Then, the FPKM values of *MdSDGs* among these samples were illustrated with heat maps by the TBtools [35]. For different abiotic stress treatments, the ‘Golden Delicious’ (*Malus×domestica*) seedlings were generated from the seeds of ‘Golden Delicious’ fruit trees which located in Horticultural Experimental Base, Northwest A&F University, Yangling, China (34°20’N, 108°24’E). We have all the permissions for using these plant materials. Three-month old seedlings were treated with various abiotic stresses including cold (4 °C for 3 h), heat (45 °C for 0.5 h), and drought (6 days). The leaf samples were frozen in liquid nitrogen and submitted to the following RNA-seq analysis with three independent biological replicates (SRS1872560). For drought treatment with different processing times, three-month old *Malus hupehensis* seedings were under drought treatment with 0, 3, 6, and 9 days. The leaf samples were frozen in liquid nitrogen and collected for RNA-seq analysis (SRX9849011). For abiotic stress treatments, each sample had three biological replicates with independent library construction and high-throughput sequencing analysis. The FPKM values of *MdSDGs* were used to reflect the gene expression levels and displayed with heat maps (Fig. 6).

### Plant materials and drought treatment

The drought treatment of ‘Golden delicious’ were conducted at Northwest A&F University, Yangling, China (34°20’N, 108°24’E). In spring, we grafted the ‘Golden delicious’ buds on *Malus hupehensis*. After scions growing up, uniform trees were selected and performed the drought treatment with 0, 2, 4, 6 and 8 days. Each treatment was set three replicates and leaf samples were frozen with liquid nitrogen for − 80 °C storage. For PEG treatment, ‘Golden delicious’ tissue cultured plants were generated from ‘Golden Delicious’ buds from plants cultured in Horticultural Experimental Base, Northwest A&F University, Yangling, China (34°20’N, 108°24’E). The buds were surface-sterilized by HgCl_2_ solution and then grown on MS medium (4.43 g/L MS salts, 30 g/L sucrose, 0.2 mg/L 6-BA, 0.2 mg/L IAA and 7.5 g/L agar, pH 6.0) under long-day condition (the light: dark cycle is 14 h: 10 h) at 25 °C for 1 month. Then, the grown-up plants were carried out rooting process on MS medium (2.22 g/L MS salts, 20 g/L sucrose, 0.5 mg/L 6-BA, 0.2 mg/L IAA and 7.5 g/L agar, pH 6.0) under long-day condition (14 h: 10 h, light: dark cycle) at 25 °C for 1 month. Then, the seedling plants were transplanted in the soil for 1 month. These plants were adopted to the hydroponic culture process in plastic containers containing 20 L of Hoagland solution for an additional month. After that, plants were treated with 20% (w/v) PEG8000 (Sigma) for 0, 2, 4, 6 and 8 h. For each treatment, three biological replicates of leaf samples were snap frozen with liquid nitrogen and stored at − 80 °C until the following qRT-PCR analysis.

### RNA extraction and real-time RT-PCR analysis

For each treatment samples, three biological replicates were performed for qRT-PCR analysis. Total RNA was extracted from the apple leaves by using CTAB method [69]. The extracted RNA was detected by electrophoresis and the concentration was determined by Nanodrop2000. The qualified RNA was subjected to reverse transcription using a Revert Aid First Strand cDNA Synthesis Kit (K1622; Thermo Fisher Scientific, Waltham, MA, USA) according to the manufacturer’s instructions. The primers were diluted in ChamQ SYBR qPCR Master Mix (Q311–02; Vazyme, NKG, CHN) and the reaction conditions were performed using an initial incubation for 30 s at 95 °C and then cycled at 95 °C/10 s and 60 °C/30 s for 40 cycles. qRT-PCR analysis was carried out with ABI StepOnePlus Real-Time PCR System (Thermo Fisher, MA, USA) using an SYBR Green-based PCR assay. The apple malate dehydrogenase gene (*MdMDH*) was used as the reference gene. All the qRT-PCR data were calculated as relative units after normalization to the reference gene as the internal control. The relative expression levels were calculated with 2^−ΔΔCT^ method. The gene-specific primers used for qRT-PCR are listed in Table S3.

### Subcellular localization assays and putative interaction network analysis of MdSDGs

To further explore the subcellular localization of MdSDG proteins, Plant-mPLoc (http://www.csbio.sjtu.edu.cn/bioinf/plant-multi/) was applied to predict the subcellular localization of MdSDGs. The full-length CDS sequences of *MdSUVH1*, *MdSUVH7*, *MdSUVH8* and *MdSUVR3* were cloned into PDONR222 entry vector and then constructed into pGWB406 expression vector by applying Gateway system. The successful constructs were transformed into *Agrobacterium* strain C58C1. Then, the positive *Agrobacterium* was infiltrated into 4-week-old *Nicotiana benthamiana* leaves together with 35S: P19 in *Agrobacterium* strain C58C1. The infected tobacco plants were further grown for 3 days at 21 °C with 16 h: 8 h (light: dark cycle) in a growth chamber. DAPI (4,6-diamidino-2-phenylindole dihydrochloride) was used to identify the nucleus. Confocal imaging was performed using an inverted Leica TCS SP8 laser scanning microscope (Leica) with a PMT detector. DAPI excitation was performed using a 405 nm solid-state laser, and fluorescence was detected at 430–470 nm. GFP excitation was performed using a 488 nm solid-state laser, and fluorescence was detected at 498–540 nm. For imaging DAPI and GFP together, excitation lines of a solid-state laser of 405 nm for DAPI and a laser of 488 nm for GFP were used alternately with line switching using the sequential scanning of the microscope. Fluorescence was detected using a 430–470 nm bandpass filter for DAPI and a 498–540 nm bandpass filter for GFP. In this way, any cross-talk and bleed-through of fluorescence were eliminated. Pinholes were adjusted to 1 airy unit for each wavelength. Images of 8 bits and 1024 × 1024 pixels were acquired using line average of 4 and pinhole of 1 airy unit. Images were post-processed using the Leica LAS X software (Version 3.7.2), ImageJ 1.46, and Adobe Photoshop 5.0 software. The experiment was repeated three times and at least three tobacco plants were infiltrated each time for each gene. The putative interaction network of MdSDGs was constructed by the STRING software (http://string-db.org/) [70]. The putative interaction network of MdSDGs was generated based on the corresponding *Arabidopsis* SDG homologs. The MdSDG IDs were correspondingly added in the Fig. S3.

## Supplementary Information

**Additional file 1: Table S1.** Characterization of SET-domain proteins (SDGs) in apple. **Table S2.** The genomic ID of SET-domain group proteins used in this study. **Table S3.** The primers of *MdSUVHs* in real-time RT-PCR analysis. **Fig. S1**. The conserved motifs of SET domain in MdSDG protein sequences. The predicted protein sequences of SET domain were extracted from each MdSDGs and then aligned by Clustal Omega (https://www.ebi.ac.uk/Tools/msa/clustalo/). According to the alignment result, the conserved motifs were displayed by WebLogo to illustrate conserved amino acid sites. **Fig. S2.** Expression patterns of *MdSDGs* among 72 dissected apple tissues. The expression levels of *MdSDGs* were extracted from the released transcriptome data among 72 dissected apple tissues (http://bar.utoronto.ca/efp_apple/cgi-bin/efpWeb.cgi). To better reflect the expression changes with heatmap, the log_2_ values of FPKM levels were adopted to generate the heatmap. **Fig. S3.** The putative interaction network of SDG proteins. The interaction network analysis was generated by STRING. The apple SDGs are in blue, and *Arabidopsis* homologous SDG proteins are in black. The line thickness indicates the confidence of the protein interaction.

## Data Availability

The apple genome datasets used in this study are available in “The Apple Genome and Epigenome” repository (https://iris.angers.inra.fr/gddh13/, version 1.1). The datasets of expression levels in different apple tissues are available in the “Apple eFP Browser” repository (http://bar.utoronto.ca/efp_apple/cgi-bin/efpWeb.cgi). The datasets of expression levels in apple fruit development and under biotic stresses are available in the AppleMDO repository (http://bioinformatics.cau.edu.cn/AppleMDO/index.php) [49]. The RNA-seq datasets of ‘Golden Delicious’ under different abiotic stresses are available in “National Center for Biotechnology Information (NCBI)” repository (SRS1872560). The RNA-seq datasets of *Malus hupehensis* seedings under drought treatments are available in NCBI repository (SRX9849011).

## Abbreviations

SDG: SET-domain group protein
GWD: Genome-wide duplication
HMTs: Histone methyltransferases
HDMs: Histone demethylases
MCScanX: Multiple Collinearity Scan toolkit
Ka: Nonsynonymous substitutions per site
Ks: Synonymous substitutions per site
RBS: Rubis-subs-bind protein
WAFB: Weak after full blooming
FPKM: Fragments Per Kilobase per Million
RdDM: RNA-directed DNA methylation
IBS: Illustrator for Biological Sequences
GSDS: Gene Structure Display Server
GFP: Green fluorescent protein
