# Supplementary material for: Genome-wide analysis of SET-domain group histone methyltransferases in apple reveals their role in development and stress responses
Source: BMC Genomics. 2021 Apr 19;22:283. doi: 10.1186/s12864-021-07596-0 (PMC8054418; doi:10.1186/s12864-021-07596-0)
Supplement: Supplementary file 1 — Additional file 1: Table S1. Characterization of SET-domain proteins (SDGs) in apple. Table S2. The genomic ID of SET-domain group proteins used in this study. Table S3. The primers of MdSUVHs in real-time RT-PCR analysis. Fig. S1. The conserved motifs of SET domain in MdSDG protein sequences. The predicted protein sequences of SET domain were extracted from each MdSDGs and then aligned by Clustal Omega (https://www.ebi.ac.uk/Tools/msa/clustalo/). According to the alignment result, the conserved motifs were displayed by WebLogo to illustrate conserved amino acid sites. Fig. S2. Expression patterns of MdSDGs among 72 dissected apple tissues. The expression levels of MdSDGs were extracted from the released transcriptome data among 72 dissected apple tissues (http://bar.utoronto.ca/efp_apple/cgi-bin/efpWeb.cgi). To better reflect the expression changes with heatmap, the log2 values of FPKM levels were adopted to generate the heatmap. Fig. S3. The putative interaction network of SDG proteins. The interaction network analysis was generated by STRING. The apple SDGs are in blue, and Arabidopsis homologous SDG proteins are in black. The line thickness indicates the confidence of the protein interaction. [file 12864_2021_7596_MOESM1_ESM.zip › Supplementary Figures.docx]

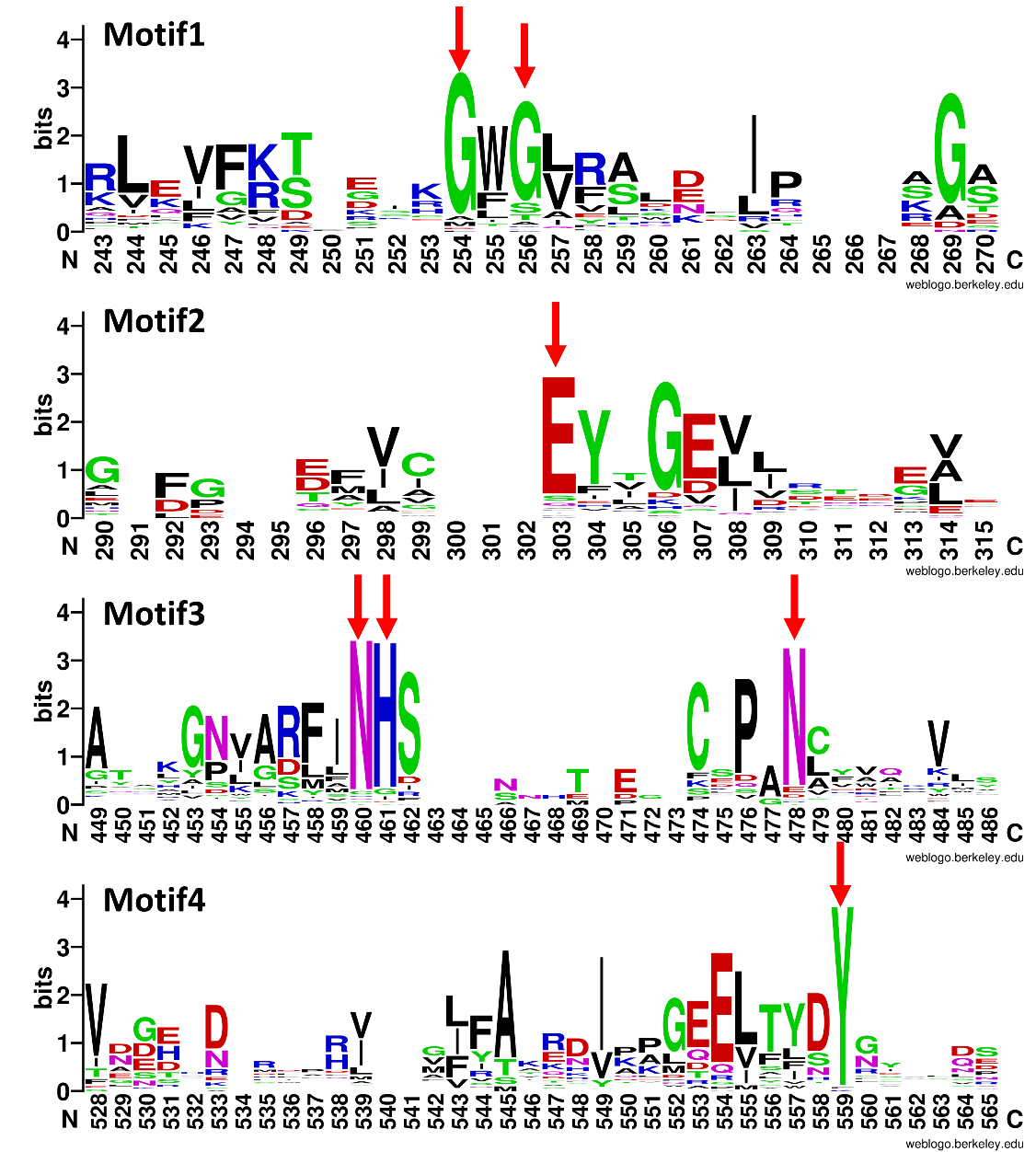


**Figure S1** **The conserved motifs of SET domain in MdSDG protein sequences.** The predicted protein sequences of SET domain were extracted from each MdSDGs and then aligned by Clustal Omega (<https://www.ebi.ac.uk/Tools/msa/clustalo/>). According to the alignment result, the conserved motifs were displayed by WebLogo to illustrate conserved amino acid sites.


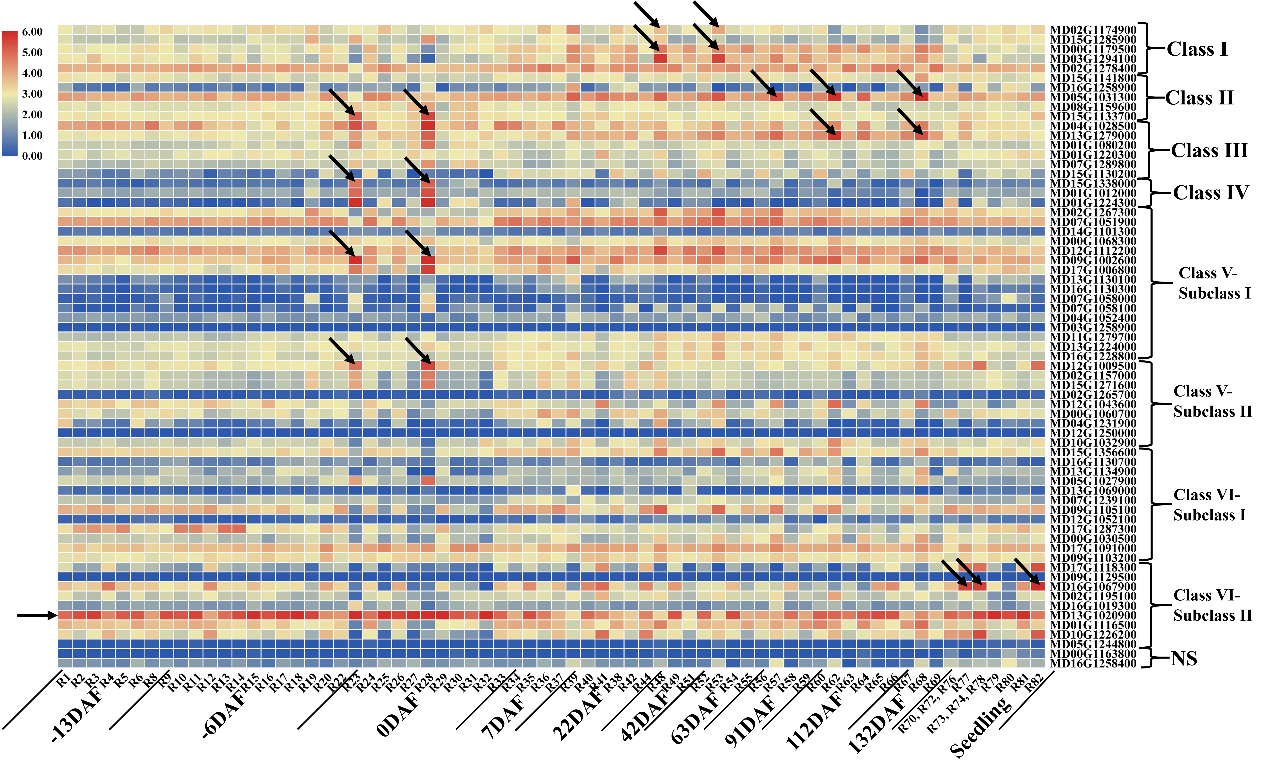


**Figure S2. Expression patterns of *MdSDGs* among 72 dissected apple tissues.** The expression levels of *MdSDGs* were extracted from the released transcriptome data in the “Apple eFP Browser” repository (<http://bar.utoronto.ca/efp_apple/cgi-bin/efpWeb.cgi>). To better reflect the expression changes with heatmap, the log_2_ values of FPKM levels were adopted to generate the heatmap.

The details of collected samples (R1-R82) are as follows: -13DAF(Days After Flowering): R1-Flower bud; R2-Pedicel; R3-Stem of inflorescence (rachis); R4-Leaves from several nodes along stem of one-year-old shoots; R5-Petiole from leaves from several nodes along stem of one-year-old shoots; R6-Skin, including tissues to 0.5 mm depth, from internodes of one-year-old shoot; R8-Apex of new shoot developing from axillary buds on 1-year-old shoots; -6DAF: R9-Flower bud; R10-Pedicel; R11-Stem of inflorescence (rachis); R12-Leaf blades from several nodes along stem; R13-Midvein from leaves from several nodes along stem; R14-Petiole from leaves from several nodes along stem; R15-Petal; R16-Pistil; R17-Receptacle; R18-Sepal; R19-Stamen; R20-Skin, including tissues to 0.5 mm depth, from shoot internodes; R22-Apex of new shoot developing from axillary buds on 1-year-old shoots, includes leaves <5 mm in length; 0DAF: R23-Anther; R24-Apex of new shoot developing from axillary buds on 1-year-old shoots; R25-Stamen filament; R26-Basal section of pistil; R27-Petal; R28-Pollen; R29-Trichome from pedicel; R30-Sepal; R31-Stigma; R32-Style; R33-Leaf, 1-5 mm from apex of new shoot; 7DAF: R34-Apex and axillary bud of new shoot, includes leaves <1 mm in length; R35-Fruit, minus seeds; R36-Seed; R37-Leaf, 1-5 mm from apex of new shoot; R38-Apex and axillary bud of new shoot, includes leaves <1 mm in length; 22DAF: R39-Embryo, dissected from seed; R40-Fruit, minus seeds; R41-Blade from more basal leaf of new shoot; R42-Seed; R44-Blade from more apical leaf of new shoot; 42DAF: R48-Embryo; R49-Fruit, minus seeds; R51-Seed coat, including some endosperm; 63DAF: R52-Apex and axillary bud of new shoot, includes leaves <1 mm in length; R53-Embryo; R54-Fruit, minus seeds; R55-Seed coat; 91DAF: R56-Apex and axillary bud of new shoot, includes leaves <1 mm in length; R57-Embryo; R58-Fruit, minus seeds; R59-Seed coat; 112DAF: R60-Apex and axillary bud of new shoot, includes leaves <1 mm in length; R62-Embryo; R63-Fruit, minus seeds and skin; R64-Seed coat; R65-Fruit skin, green; R66-Fruit skin, purple; 132DAF: R67-Apex and axillary bud of new shoot, includes leaves <1 mm in length; R68-Embryo; R69-Fruit, minus seeds and skin; Open-pollinated seedlings: R70-Shoot apex, includes leaves <1 mm in length; R72-Shoot apex, includes leaves <1 mm in length; R73-Apical leaf; R74-Apical leaf; R76-Shoot apex, includes leaves <1 mm in length; R77-Cotyledon; R78-Leaf, 1 to 5 mm in length from apex; R79-Hypocotyl; R80-Root; R81-Main stem above cotyledon, includes petioles; R82-Leaf blade.


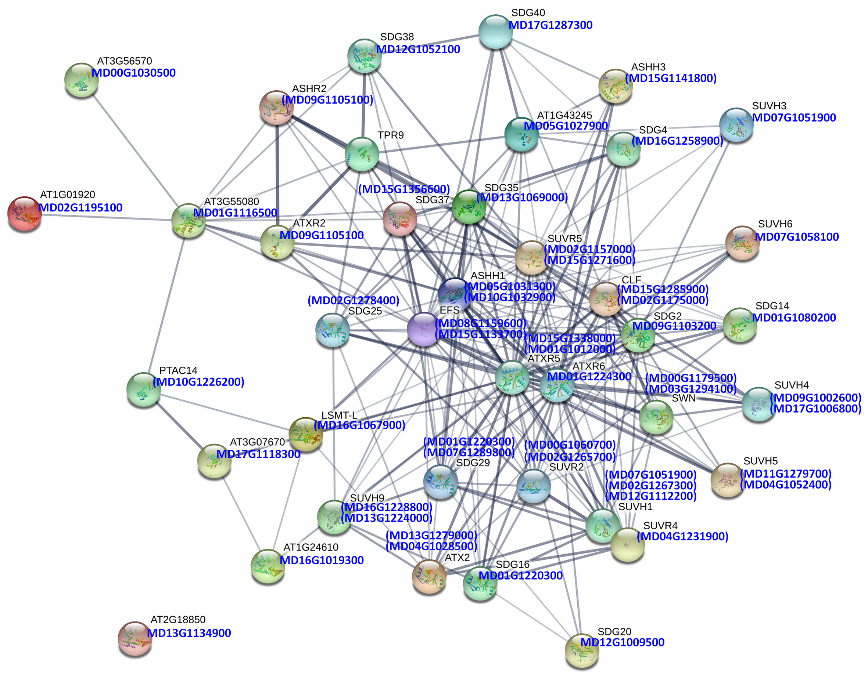


**Figure S3. The putative interaction network of SDG proteins.** The interaction network analysis was generated by STRING. The apple SDGs are in blue, and *Arabidopsis* homologous SDG proteins are in black. The line thickness indicates the confidence of the protein interaction.
